# How Reliable Are Gene Expression-Based and Immunohistochemical Biomarkers Assessed on a Core-Needle Biopsy? A Study of Paired Core-Needle Biopsies and Surgical Specimens in Early Breast Cancer

**DOI:** 10.3390/cancers14164000

**Published:** 2022-08-18

**Authors:** Hani Saghir, Srinivas Veerla, Martin Malmberg, Lisa Rydén, Anna Ehinger, Lao H. Saal, Johan Vallon-Christersson, Åke Borg, Cecilia Hegardt, Christer Larsson, Alaa Haidar, Ingrid Hedenfalk, Niklas Loman, Siker Kimbung

**Affiliations:** 1 Division of Oncology, Department of Clinical Sciences, Lund University, SE-223 81 Lund, Sweden; 2 Department of Hematology, Oncology and Radiation Physics, Lund University Hospital, SE-221 85 Lund, Sweden; 3 Department of Surgery and Gastroenterology, Skåne University Hospital, SE-214 28 Malmö, Sweden; 4 Department of Clinical Genetics and Pathology, Lund University Hospital, SE-221 84 Lund, Sweden; 5 Division of Translational Cancer Research, Department of Laboratory Medicine, Lund University, SE-223 81 Lund, Sweden; 6 Department of Surgery and Oncology, Hallands Hospital, SE-302 33 Halmstad, Sweden

**Keywords:** genomic profiling, breast cancer, core-needle biopsy, immunohistochemistry

## Abstract

**Simple Summary:**

This study aims to assess the reliability of using a core-needle biopsy (CNB) for preoperative tumor characterization using gene expression analysis and conventional immunohistochemistry (IHC) analysis for clinical biomarkers in early breast cancer. Obtaining a preoperative CNB is a standard procedure in the evaluation of a primary breast cancer, with previous studies suggesting that it can be considered trustworthy to perform immunohistochemical analysis on CNB samples. However, little research has been carried out to evaluate whether gene expression-based biomarker assessment can be carried out reliably on the preoperative CNB. This information is important as genomic profiling is gaining ever-increasing importance in the treatment of early breast cancer.

**Abstract:**

In early breast cancer, a preoperative core-needle biopsy (CNB) is vital to confirm the malignancy of suspected lesions and for assessing the expression of treatment predictive and prognostic biomarkers in the tumor to choose the optimal treatments, emphasizing the importance of obtaining reliable results when biomarker status is assessed on a CNB specimen. This study aims to determine the concordance between biomarker status assessed as part of clinical workup on a CNB compared to a medically untreated surgical specimen. Paired CNB and surgical specimens from 259 patients that were part of the SCAN-B cohort were studied. The concordance between immunohistochemical (IHC) and gene expression (GEX) based biomarker status was investigated. Biomarkers of interest included estrogen receptor (ER; specifically, the alpha variant), progesterone receptor (PgR), Ki67, HER2, and tumor molecular subtype. In general, moderate to very good correlation in biomarker status between the paired CNB and surgical specimens was observed for both IHC assessment (83–99% agreement, kappa range 0.474–0.917) and GEX assessment (70–97% agreement, kappa range 0.552–0.800), respectively. However, using IHC, 52% of cases with low Ki67 status in the CNB shifted to high Ki67 status in the surgical specimen (McNemar’s *p* = 0.011). Similarly, when using GEX, a significant shift from negative to positive ER (47%) and from low to high Ki67 (16%) was observed between the CNB and surgical specimen (McNemar’s *p* = 0.027 and *p* = 0.002 respectively). When comparing biomarker status between different techniques (IHC vs. GEX) performed on either CNBs or surgical specimens, the agreement in ER, PgR, and HER2 status was generally over 80% in both CNBs and surgical specimens (kappa range 0.395–0.708), but Ki67 and tumor molecular subtype showed lower concordance levels between IHC and GEX (48–62% agreement, kappa range 0.152–0.398). These results suggest that both the techniques used for collecting tissue samples and analyzing biomarker status have the potential to affect the results of biomarker assessment, potentially also impacting treatment decisions and patient survival outcomes.

## 1. Introduction

Minimally invasive tumor biopsies are vital in the preoperative workup of breast lesions. Obtaining a preoperative core-needle biopsy (CNB) on suspected breast lesions is a standard procedure in the primary evaluation of breast cancer and can be used to accurately differentiate invasive disease from *in situ* lesions with a high sensitivity and specificity [1,2,3,4,5,6,7]. Neoadjuvant systemic treatment which is administered before definitive breast surgery is also becoming more widely implemented for a significant number of patients diagnosed with primary breast cancer since it offers the possibility of breast conservation by downsizing, without compromising prognosis [8,9]. In addition, preoperative therapy offers the unique opportunity to evaluate tumor response allowing for rational treatment modification, if necessary, following surgery [10,11,12,13].

The choice of systemic therapy in the preoperative setting is dependent on tumor biomarker classification performed on the preoperative CNB together with clinical and radiological tumor staging. Furthermore, the type of medical treatment offered to patients unfit for surgical excision of the tumor is also based on the biomarker status classification determined on a CNB. Although many local and international guidelines permit biomarker assessment on either CNB or surgical specimens, variability in biomarker status between paired CNB and surgical specimens, with the potential to impact choice of therapy have been previously reported [14,15].

Immunohistochemistry (IHC), including *in situ* hybridization (ISH) for equivocal human epidermal growth receptor-2 (HER2) cases, is the method of choice for determining the status of the conventional histopathological biomarkers in breast cancer including estrogen receptor (ER), progesterone receptor (PgR), HER2, and the proliferation marker Ki67 [16,17,18]. These four biomarkers carry prognostic and treatment predictive information and are used in combination with other clinicopathological factors such as Nottingham histologic grade (NHG), nodal status, and tumor size for risk stratification and therapy selection. Other analytical methods including gene expression profiling (GEX) have been developed for assessing these conventional biomarkers [19,20,21,22,23] and other novel multigene prognostic and predictive signatures. In fact, some GEX-based tests are already approved by regulatory agencies to support prognostic and treatment predictive decisions in routine clinical practice [24,25,26,27,28,29]. However, it has not been well established whether these GEX-based assays produce reliable or similar estimations of biomarkers when performed on a CNB compared with a surgical specimen. A better understanding of the analytical validity of gene expression-based assays is necessary considering their broad development for routine clinical implementation and the common practice in research studies where missing data for a biomarker is completed by supplementing with data collected using a different technique, if available, e.g., IHC and GEX.

This study aimed to determine the concordance of biomarker status assessed using IHC and mRNA sequencing (RNAseq) in paired, untreated preoperative CNB and surgical specimens from a consecutive population-based cohort of 259 patients with early breast cancer.

## 2. Materials and Methods

### 2.1. Study Population and Sample Collection

The study is based on the Sweden Cancerome Analysis Network-Breast (SCAN-B) cohort which is an ongoing multicenter population-based observational study that was initiated in September 2010 with the purpose of analyzing breast tumors using next generation genomic technologies to facilitate translational research (ClinicalTrials.gov identifier NCT02306096). As of 30 June 2022, over 18,600 patients were enrolled in SCAN-B, representing about 85% of eligible patients in the catchment area. Sample collection and processing within SCAN-B has been described previously [30,31]. Briefly, for cases included in this sub-study, the preoperative CNB sample was obtained by a radiologist using ultrasound guidance and at least two separate cores were taken for histopathology and GEX analyses by RNAseq respectively. At the subsequent surgical procedure, tumor samples were again collected by the pathologist during the postoperative workup for histopathological assessment and RNAseq. Written informed consent was collected from all included patients. The SCAN-B study was approved by the Regional Ethical Review Board of Lund (registration numbers 2009/658, 2010/383, 2012/58, 2013/459 2014/521, 2015/277, 2016/944), the county governmental biobank center and the Swedish Authority for Privacy Protection (registration number 364-2010).

**Figure 1:**
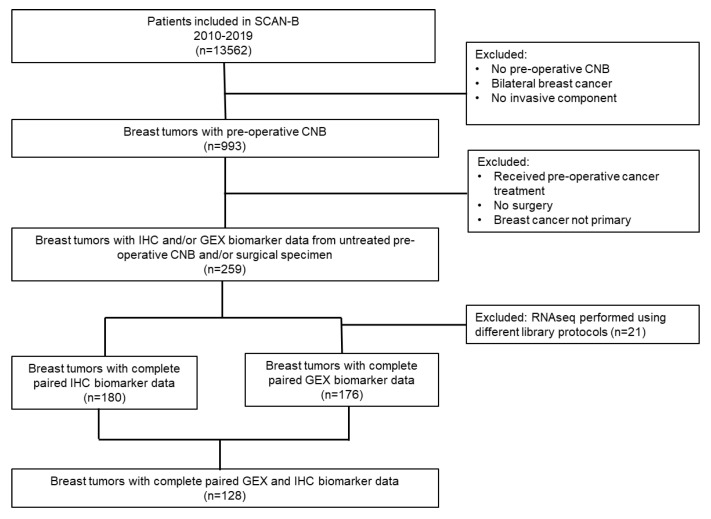
Flowchart showing the selection of the study specific cohort (*n* = 259) from the SCAN-B cohort.

Selection of patients and tumors for this sub-study is presented in Figure 1. Women presenting with early-stage breast cancer treated primarily with surgery, and who had received no preoperative medical treatment were eligible. Patients with bilateral breast cancers were excluded. Patients were further selected based on the availability of paired CNBs and surgical specimens with the corresponding IHC and/or GEX data. In total, 259 patients were eligible for inclusion in our analyses to assess the concordance of the biomarker status and differences in the transcriptome between CNBs and paired surgical specimens as presented in this study.

### 2.2. Biomarker Assessment Using Immunohistochemistry (IHC)

IHC analyses of biomarkers were performed as per routine clinical practice at five different local pathology departments (Malmö, Kristianstad, Helsingborg, Lund, and Halmstad) and reported in the medical records of the patients. Study-specific re-assessment of IHC biomarkers was not performed, to reflect the concordance levels in routine clinical practice. Other patient and tumor characteristics were obtained from the Swedish National Quality Register for Breast Cancer (NKBC) [8] and missing data were collected directly from patient records, wherever possible.

Dichotomized variables for the conventional IHC biomarkers were used in statistical analyses as follows: positive vs. negative for ER, PgR, and HER2; high vs. low for Ki67. The cutoff used for ER and PgR expression was ≥10% for positive tumors, consistent with the national Swedish guidelines [32]. For HER2 interpretation, tumors with IHC scores between 0–1+ and scores 3+ were classified as HER2-negative and HER2-positive respectively. Tumors showing IHC staining scores of 2+ were further tested using fluorescence *in situ* hybridization (FISH) or silver-enhanced *in situ* hybridization (SISH), and if *HER2* gene amplification was detected, the tumor was classified as HER2-positive. Ki67 index was determined by counting at least 200 cells within areas with the highest observed Ki67 expression, i.e., “hotspots”. Both standard microscopic examination and digital image analysis were acceptable methods. Surrogate molecular subtypes were determined following the 2013 St. Gallen consensus with the slight modification of using a Ki67 low expression cutoff of <20% instead of <14% as specified in the consensus [33]. At the time of this study, before adopting the thresholds defined by the International Ki67 Working Group [34], Swedish quality assurance program guidelines recommended that each laboratory calibrate a Ki67 cutoff yearly such that one third of 100 consecutive cases are Ki67-high. The cutoffs for high Ki67 expression range between 17–31% for the local pathology departments for the SCAN-B study. A cutoff of 20% was selected for use in the current analyses based on the expected ratio of luminal A-like/luminal B-like tumors in our cohort [32,33].

### 2.3. Gene Expression Analysis (GEX)

The RNAseq workflow for all tumors included in the SCAN-B cohort has been described in detail previously [30,31]. Briefly, a preoperative CNB is taken by a radiologist using ultrasound guidance and placed directly in RNAlater reagent. Following surgery, routine assessment of the surgical specimen is performed by a pathologist who selects a piece of tumor-cell enriched fresh specimen(s) and preserves it directly in RNAlater reagent. Fresh tumor specimens (CNBs and surgical specimens) in RNAlater are sent directly after collection from all participating clinical sites to the SCAN-B laboratory for central processing with handling standards that meet or exceed recommendations of the Breast International Group (BIG). Each tumor specimen is weighed, and when possible, partitioned into three parts as follows: one piece for isolation of nucleic acids and protein; one adjacent piece to be fixed in formalin and used for construction of a tissue microarray (TMA); and any remaining tissue is stored frozen for future use. The TMA is used for histopathological assessment and estimation of tumor cellularity and the TMA also serves as a research resource. Nucleic acid extraction for RNAseq is always prioritized. Nucleic acids (and protein) are isolated from the tumor specimen using the AllPrep method (Qiagen). RNA and DNA quality control is performed by NanoDrop spectrophotometry and BioAnalyzer (Agilent) or Caliper LabChip XT (PerkinElmer) capillary gel analysis. Customized protocols for RNA-seq using 1 μg of starting total RNA were developed and automated for a high-throughput workflow. The complete methods and protocols are described in the full details in [30]. For inclusion in this study, an RNA quality score (Rin) ≥7 was required for all samples.

The multi-gene RNAseq based classifiers for predicting the status of the standard breast cancer biomarkers (ER, PgR, HER2, and Ki67) developed by the SCAN-B work group were implemented in this study [23]. These gene expression-based classifiers were trained on consensus histopathology labels and displayed substantial agreements to conventional IHC in the validation cohort. PAM50 molecular subtypes were assigned by the nearest centroid classification method as previously described [29]. Finally, an exploratory whole transcriptome paired significance analysis of microarray (SAM) analysis [35] was implemented to identify significant differentially expressed genes between paired CNBs and surgical specimens (with significance parameters, FDR q-value = 0). This was followed by gene set enrichment analyses (GSEA) [36] to identify statistically significant enriched biological processes, pathways, and cancer hallmarks between the untreated paired CNBs and surgical specimens.

### 2.4. Statistical Analysis

The statistical significance for the concordance of biomarker expression between the paired CNB and surgical specimen and between different analytical methods was evaluated using both Cohen’s kappa and McNemar’s tests, wherever applicable. Two-sided statistical analyses were performed and *p* < 0.05 was considered to be statistically significant. Statistical analyses were performed using SPSS statistics version 27.

## 3. Results

### 3.1. Distribution of Patient and Tumor Characteristics

The distribution of patient and tumor characteristics among the 259 patients is presented in Table 1 and Supplementary Table S1. The median age at diagnosis was 63 years (range 27–88 years). 46%, 12%, and 81% of patients presented with T1 tumors, NHG I tumors, and negative lymph nodes, respectively. The majority (68%) of patients were postmenopausal women. The distribution of biomarkers assessed by IHC and GEX for CNBs and surgical specimens is presented in Supplementary Table S1. One hundred and eighty (69%) paired CNBs and surgical specimens had complete data for all biomarkers by IHC, whilst 176 (68%) pairs had complete GEX classification for all biomarkers.

### 3.2. Concordance of Biomarker Status Analysed Using the Same Method (IHC or GEX) in Paired Core-Needle Biopsy and Surgical Specimen

**Figure 2:**
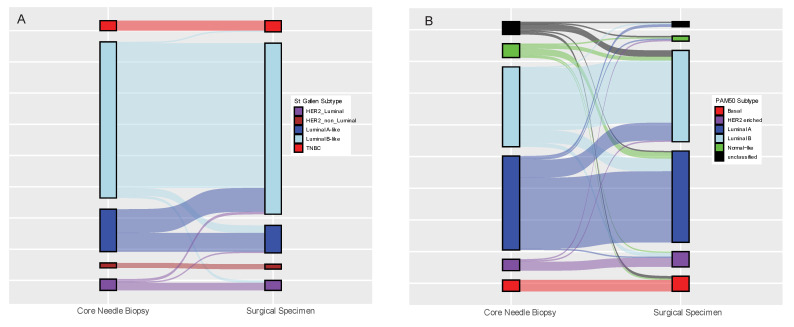
Concordance of St Gallen subtypes (**A**) and PAM50 subtypes (**B**) between paired core-needle biopsies and surgical specimens.

First, the biomarker status of the paired CNB and surgical specimen were compared for each analytical method as reported in Table 2. The RNA yield and quality obtained from CNBs in our study were excellent as sufficient RNA and of good quality was achieved for more than 98% of the samples. Note that cases for which different library protocols were used for RNAseq for the paired CNB and surgical specimen were excluded from the GEX concordance analysis presented herein. The pairwise agreements for IHC evaluation were high: ER 98.9% (kappa 0.92), PgR 92.3% (kappa 0.78), HER2 97.2% (kappa 0.79), Ki67 82.9% (kappa 0.47), and St. Gallen subtype 82.8% (kappa 0.61). The pairwise agreements of the GEX biomarkers were relatively lower compared to IHC, except for Ki67, which displayed a higher agreement by GEX: ER 88.1% (kappa 0.61), PgR 85.2% (kappa 0.59), HER2 97.2% (kappa 0.80), Ki67 86.4% (kappa 0.70), and PAM50 subtype 69.9% (kappa 0.55). However, we observed interesting trends in the discordance of some biomarkers between the CNB and the paired surgical specimen. Using IHC, 31 out of 181 tumors (17.1%) displayed discordant Ki67 status with 23/31 (72%) shifting from low expression in the CNB to high expression in the surgical specimen (Table 2, McNemar’s *p* = 0.011). This shift in Ki67 corresponded to 60% (19/34) Luminal A-like to Luminal B-like subtype conversions between CNB vs. surgical specimens (Figure 2A); however, the overall shift in St. Gallen subtypes between CNBs and surgical specimens was not statistically significant (McNemar’s *p* = 0.067). Similarly, using GEX, 16 out of the 21 (76%) ER discordant cases shifted from negative ER status in the CNB to positive ER status in the surgical specimen (McNemar’s *p* = 0.027). Ki67 status was discordant in 24 cases, 20 (83%) of which shifted from low expression in the CNB to high expression in the surgical specimen (McNemar’s *p* = 0.002). Interestingly, 9/10 (90%) tumors which were unclassifiable into any molecular subtype using GEX in the CNB were successfully classified into one of the five intrinsic molecular subtypes in the surgical specimen and Luminal A to Luminal B shifts were more prevalent (Figure 2B, McNemar´s *p* = 0.168).

### 3.3. Concordance between Biomarker Status Analyzed by GEX versus IHC for CNB or Surgical Specimens, Respectively

Next, we investigated the agreement between IHC and GEX biomarker status separately for CNBs and surgical specimens (Table 3). The respective pairwise agreements for IHC vs. GEX performed on CNBs were ER 85.7% (kappa 0.40), PgR 80.8% (kappa 0.48), HER2 96.6% (kappa 0.71), Ki67 47.6% (kappa 0.15), and subtype 57.3% (kappa 0.26). Similarly, the respective pairwise agreements for IHC vs. GEX performed on surgical specimens were ER 91.5% (kappa 0.64), PgR 86.3% (kappa 0.59), HER2 93.5% (kappa 0.63), Ki67 55.3% (kappa 0.21), and subtype 61.6% (kappa 0. 40). Notably, a tendency to switch classification from positive to negative ER, high to low Ki67 and from Luminal B to Luminal A subtype was consistently observed when comparing IHC with GEX classification for both CNBs and surgical specimens (Table 3). Specifically, 23/24 (96%) CNBs showing discordant ER status were classified as ER positive by IHC and ER negative by GEX, 84/87 (97%) of CNBs were discordant for Ki67; classified as high by IHC and low by GEX, and 48/105 (45.7%) of samples classified as Luminal B-like by IHC were classified as Luminal A by GEX. Likewise, 18/18 (100%) surgical specimens showing discordant ER status were all classified as ER positive by IHC and ER negative by GEX, 99/99 (100%) of surgical specimens were discordant for Ki67 being classified as high by IHC and low by GEX, and 63/149 (42.3%) of samples classified as Luminal B-like by IHC were classified as Luminal A by GEX.

### 3.4. Differential Global Gene Expression Profiles of Paired CNBs and Surgical Specimens

**Figure 3:**
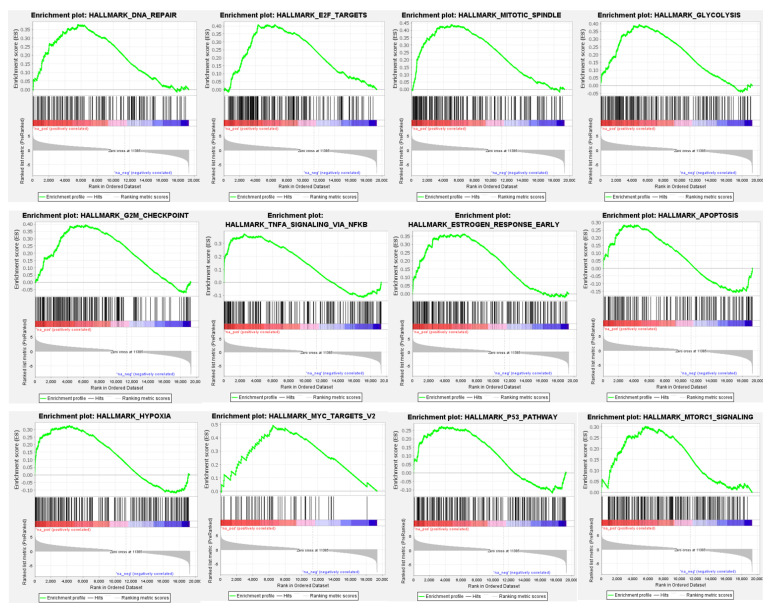
Gene Set Enrichment Analysis (GSEA) for RNAseq profiles between paired CNBs and surgical specimens. Examples of histograms showing the distribution of the Hallmark of cancer pathways enriched among genes showing significantly higher expression in CNBs compared with the paired surgical specimens. Refer to Supplementary Table S3 for the full list of the enriched Hallmark of cancer pathways among the genes with higher expression in CNBs compared with surgical specimens.

**Figure 4:**
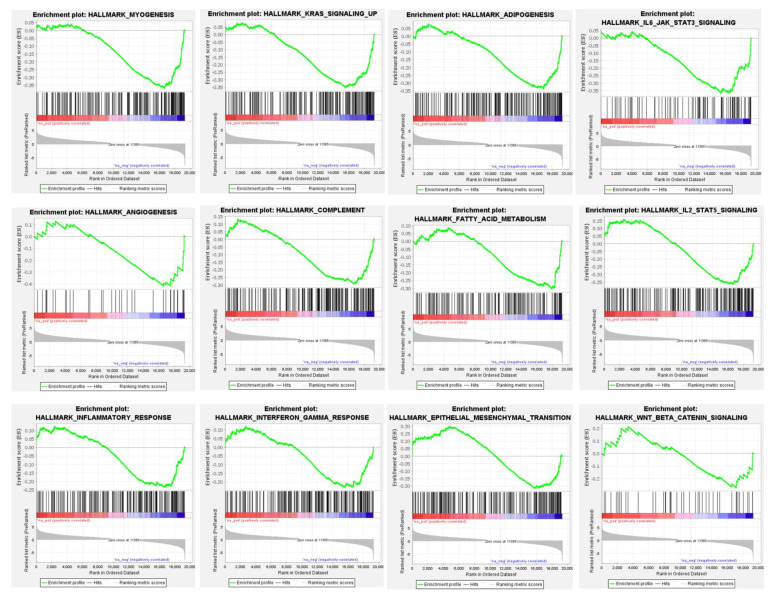
Gene Set Enrichment Analysis (GSEA) of RNAseq profiles between paired CNBs and surgical specimens. Examples of histograms showing the distribution of the Hallmark of cancer pathways enriched among genes showing significantly lower expression in CNBs compared with the paired surgical specimens. Refer to Supplementary Table S3 for the full list of the enriched Hallmark of cancer pathways among the genes with lower expression in CNBs compared with surgical specimens.

Three hundred and eleven (311) genes were significantly differentially expressed between paired CNB and surgical specimens; 172 with higher and 139 with lower relative expression in CNBs compared with the paired surgical specimens (FDR = 0, Supplementary Table S2). Significantly enriched hallmark of cancer genesets among the genes showing higher expression among CNBs included DNA repair, cell cycle, TNF-alpha signaling, hypoxia, p53 pathway, mTOR signaling, mitotic spindle, estrogen response, notch signaling, and apoptosis, among others (Figure 3 and Supplementary Tables S3 and S4). Gene sets associated with myogenesis, KRAS signaling, angiogenesis, IL6 JAK STAT3 signaling, epithelial mesenchymal transition, and fatty acid metabolism were found to be enriched among genes with decreased expression among CNBs (Figure 4 and Supplementary Tables S3 and S4). The GSEA results were consistent for the genesets included in the hallmark of cancer and Reactome databases (Supplementary Tables S3 and S4).

## 4. Discussion

Several factors can impact the accuracy of analyses performed on preoperative CNBs, such as the size of the core-needle used, the number of samples taken, tumor size, and the experience and expertise of the radiologist performing the procedure. In this real-life study, these factors may have varied for the different patients, and the analyses presented herein focus instead on investigating how reliable a preoperative CNB is in routine clinical practice when determining the status of conventional breast cancer prognostic and treatment predictive biomarkers using IHC and GEX respectively.

The surgical specimen is usually considered as the gold standard to obtain information about the status of different biomarkers for a breast cancer. The surgical specimen allows for an overview of the whole tumor area of that tissue section, which is essential for the assessment of the surgical margin. It also allows for the estimation of invasive areas, presence of *in situ* cancer, and the spatial expression of biomarkers in the tumor. The area of the tumor that is represented in a tissue section is limited but is nevertheless larger compared to the area represented by a CNB, where there is a greater risk that the material taken does not fully represent the entire nature of the tumor, e.g., in terms of tumor heterogeneity.

In this study, we observed moderate to very good concordance of biomarkers between paired preoperative CNB and surgical specimen when the same technique (IHC or GEX) was used for biomarker assessment. The concordance for IHC biomarkers observed in this study is similar to results reported in previous studies; ER (68–100%), PgR (71–89%), HER2 (60–100%), Ki67 (80–82%) [37,38,39,40,41,42,43,44,45]. Recent research also suggests modest to strong correlation of the gene expression patterns between paired CNBs and surgical specimen [46,47], in line with our results. A reassuring finding in this study was the consistently high level of agreement in the HER2 status between CNB and surgical specimens for both IHC and GEX (97.24% and 97.16%, respectively). HER2 status is essential in deciding whether to provide anti-HER2 targeted treatments in breast cancer [48,49]. It must be highlighted that because the multigene classifiers for the conventional breast cancer biomarkers (ER, PgR, Ki67, and HER2) used in this study are trained on histopathological labels, it is not surprising that tumors assigned a positive HER2_GEX_ status are not necessarily classified as HER2-enriched by the conventional PAM50 molecular subtype classifier.

Our study results also show that for most of the biomarkers, the concordance of GEX-based classifiers between the CNBs and surgical specimen were inferior to the concordance observed for the standard IHC. Tumor heterogeneity and sampling error are factors which can be accounted for when a biomarker is assessed using IHC by a trained pathologist. On the other hand, results of transcriptional profiling of tumors using techniques like bulk RNAseq represent the average gene expression levels in the sample, including non-malignant cells found in the sample, which may impact the classification of samples. The development of methods which ensure that the CNB collected for biomarker analyses is indeed rich in tumor cells can potentially improve the analytical validity of GEX profiling in CNBs. An example of a method under experimentation at our local pathology department is obtaining a punch biopsy from the surgical specimen and studying the tumor cellularity in the borders of the punch by H&E staining to ensure that the punch biopsy was taken from a section of the tumor with sufficient malignant cells, prior to extracting RNA for gene expression analysis. Tumor cellularity in the borders of CNBs will also be investigated, although this will be a more complex process.

The multigene classifiers have been shown to provide valuable added clinical information, particularly for IHC ER-positive tumors which were predicted to display ER-negative properties according to the GEX classifier [23]. Specifically, tumors which were classified discordantly as ER_IHC_ positive and ER_GEX_ negative by this multigene classifier showed an inferior survival compared to tumors concordantly classified as ER positive [23]. In this sub-cohort, we observed similar trends in the concordance levels between the histopathological labels (IHC) and multigene signatures for ER, PgR, HER2, and Ki67; with the highest agreement observed for ER expression and the lowest agreement for Ki67, regardless of the sample type (CNBs or surgical specimens). Interestingly, a pattern for the discordance overwhelmingly favoring a change from positive ER-status by IHC to negative ER status by GEX in both the CNBs (23/24) and surgical specimen (18/18), respectively was noted. Moreover, in this study, the majority of the IHC ER positive cases discordantly classified as ER negative by GEX (18/23 on CNB, 12/18 on the surgical specimen) also had a high (>50%) percentage for ER-staining on IHC. This suggests that the ER status determined by GEX might better reflect the endocrine responsiveness of the tumor, adding clinically relevant information for the management of some tumors immunohistochemically classified into the luminal subtype.

Despite GEX-based biomarkers being incorporated into Swedish and international guidelines to help predict the benefit from adjuvant chemotherapy, current guidelines on neoadjuvant chemotherapy suggest that there is insufficient evidence for using genomic profiles in a neoadjuvant setting [50,51]. Remarkably, in this study, the GEX-classifier for Ki67 showed better concordance between the CNB and the surgical specimen when estimating the proliferative activity of the tumor, a marker where the immunohistochemical assessment is notoriously troublesome. This finding suggests that GEX may serve as a companion assay to help differentiate the more highly proliferative luminal B-like tumors from luminal A-like tumors in CNBs. This consideration is particularly interesting for ER positive tumors when IHC yields ambiguous results for risk prediction [52]. The significant discordance between CNB and surgical specimen in Ki67_IHC_ status when a similar technique was used for biomarker analysis (IHC or gene expression) reported in this study is in line with previous research on this biomarker [44]. However, we observed that when comparing Ki67 determined by IHC with Ki67 determined using GEX, almost all (>97%) discordant cases were classified as low Ki67 by GEX, suggesting a less aggressive tumor phenotype with the possibility of treatment de-escalation. The International Ki67 in Breast Cancer Working Group (IKWG) has suggested that the clinical utility of this biomarker should be limited to prognosis assessment in early-stage breast cancer which is ER-positive and HER2-negative. The report also raises the issue of lacking reproducibility of this biomarker [52]. Our results continue to shine light on the limitation of only using IHC for biomarker assessment in the preoperative setting which may not be sufficient to distinguish the more highly proliferative luminal B-like tumors from the luminal A-like tumors, a distinction which may change the recommendation on whether to pursue neoadjuvant chemotherapy or not. In the clinical setting, NHG is sometimes included as an adjunct in unclear cases of proliferation. In our study, the large extent of discordance between GEX and IHC when evaluating Ki67 status and molecular subtype may partly be explained by the fact that the IHC assessment for Ki67 was performed in different pathology labs, with different Ki67 cutoffs, whilst a single cutoff was used in this study to dichotomize Ki67 status. This does however only emphasize the poor analytical validity of this biomarker, and the lack of standardization in the analysis of this biomarker as previously suggested by the International Ki67 working group [34]. Recent efforts by the IKWG suggests that digital solutions may be required to address this issue. Using digital image analysis, 30 paired CNB and surgical breast cancer specimens were compared, and no significant difference was observed in the digitally assessed Ki67 index between serial sections. However, a systematic discrepancy between CNB and corresponding whole sections was observed—CNBs yield higher Ki67 scores (possibly due to pre-analytical factors including more standard and prompt tissue handling, fixation time, etc.). Based on these results, IKWG suggests that Ki67 IHC tested on a CNB should be preferred to excision specimens in clinical decision-making, because doing so will preclude many pre-analytical factors [52]. However, this will require that the CNB specimen collected is truly representative of the tumor. New studies comparing Ki67 status between GEX and IHC with standardized immunohistochemical Ki67 analysis are therefore recommended, and alternative ways of assessing tumor proliferation should be considered.

A further consideration regarding Ki67 is the use of this biomarker to assess treatment response, where a relative reduction of Ki67 expression levels of the tumors after brief exposure to treatment may be interpreted as a good treatment response and is associated with a favorable long-term outcome. The results of the clinical trial POETIC aiming to assess the benefit of pre- and perioperative aromatase inhibitor (AI) treatment on the clinical outcome in operable breast cancer are strongly indicative of a prognostic value of the relative Ki67 reduction [53]. In the POETIC trial, the Ki67 levels were measured on baseline, and 2 weeks after the initiation of treatment with an Aromatase Inhibitor to correlate Ki67 changes to clinical outcome. By performing comparison on the global transcriptome level in the present study, we observed significant differences in several prognostic and cancer treatment predictive genes and gene sets in this treatment naïve cohort. These results illustrate that substantial variation in the expression of genes associated with important biological processes/pathways likely related to both tumor intrinsic factors, differences in the composition of the tumor microenvironment, and the time between tissue sampling to fixation in preservative solution (RNAlater) exists even when comparing treatment-naïve CNB vs. surgical specimens. The observation that technical variations can potentially impact decisions regarding choice of therapy continues to emphasize the importance of robust validation for every step in the clinical diagnostic process. Caution must also be exercised when interpreting treatment-induced changes in genes/biomarkers. Ideally, the analytical reproducibility should be investigated for each biomarker wherever possible and differences associated with sample heterogeneity and technical variation should be taken into consideration when interpreting data about treatment-induced changes in biomarkers, especially for neoadjuvant studies and particularly in the so called window-of-opportunity studies where a baseline CNB is often compared with either an on-treatment CNB or surgical specimen and may actually be the only biomarker used to assess a potential treatment effect.

## 5. Conclusions

When using a preoperative CNB to determine the biomarker status of a tumor using IHC or GEX, the limitations of both methods should be acknowledged to correctly reflect the nature of the tumor, and if possible, investigated for new/uncharacterized biomarkers. The proliferative status of the tumor should be interpreted with caution when determined immunohistochemically using Ki67 expression, especially in the ER+/HER2− group. Moreover, the endocrine responsiveness of a tumor as determined by its immunohistochemical ER status is an aspect where complementing with a GEX-based assay may add relevant clinical value. Future studies should determine ways to improve the quality of sample collection and the reproducibility of the results of biomarkers assessed in CNBs in the clinic to ensure that patients are always provided the best treatments and spared the toxicities of ineffective treatments.

## Figures and Tables

**Table 1:** Distribution of patient and tumor characteristics.

Characteristic		N (%)
**Age**	Years	63 (27–88) *
**Tumor Stage (T)**		
T1	Tumor ≤ 20 mm	120 (46%)
T2	Tumor > 20 mm but ≤ 50 mm	122 (47%)
T3	Tumor > 50 mm	12 (5%)
T4	Tumor of any size with direct extension to chest wall or skin	1 (0.4%)
Missing		4 (2%)
**Nodal Status**		
N0	No cancer in regional nodes	210 (81%)
N+	Cancer in regional nodes	49 (19%)
**Nottingham Histological Grade (NHG)**		
Grade 1	Well differentiated	31 (12%)
Grade 2	Moderately differentiated	128 (49%)
Grade 3	Poorly differentiated	91 (35%)
Missing		9 (3%)

* Median (range).

**Table 2:** Concordance of biomarker status between paired preoperative CNB and surgical specimen assessed using IHC and GEX, respectively.

Biomarker (IHC)	CNB/Surgical Specimen	N	% Agreement	Kappa	McNemar’s *p*
**ER** (n = 183)	Pos/Pos	169	98.91%	0.917	1.000
	Neg/Neg	12			
	Pos/Neg	1			
	Neg/Pos	1			
**PgR** (n = 181)	Pos/Pos	134	92.27%	0.776	0.057
	Neg/Neg	33			
	Pos/Neg	3			
	Neg/Pos	11			
**HER2** (n = 181)	Pos/Pos	10	97.24%	0.785	1.000
	Neg/Neg	166			
	Pos/Neg	3			
	Neg/Pos	2			
**Ki67** (n = 181)	High/High	129	82.87%	0.474	**0.011**
	Low/Low	21			
	High/Low	8			
	Low/High	23			
**St Gallen Subtype** (n = 180)	LumA/LumA	15	82.78%	0.612	0.067
	LumB/LumB	116			
	HER2/HER2	10			
	TNBC/TNBC	8			
	Discordant subtype	31			
**Biomarker (GEX)**	**CNB/Surgical Specimen**	**N**	**% Agreement**	**Kappa**	**McNemar’s *p***
**ER** (n = 176)	Pos/Pos	133	88.07%	0.606	**0.027**
	Neg/Neg	22			
	Pos/Neg	5			
	Neg/Pos	16			
**PgR** (n = 176)	Pos/Pos	122	85.23%	0.588	0.076
	Neg/Neg	28			
	Pos/Neg	8			
	Neg/Pos	18			
**HER2** (n = 176)	Pos/Pos	11	97.16%	0.800	0.063
	Neg/Neg	160			
	Pos/Neg	0			
	Neg/Pos	5			
**Ki67** (n = 176)	High/High	48	86.36	0.699	**0.002**
	Low/Low	104			
	High/Low	4			
	Low/High	20			
**Pam50 Subtype** (n = 176)	LumA/LumA	56	69.89%	0.552	0.168
	LumB/LumB	49			
	HER2/HER2	7			
	Basal/Basal	9			
	Normal-like/Normal-like	1			
	Unclassified/Unclassified	1			
	Discordant subtype	53			

**Table 3:** Concordance of biomarker status assessed by IHC compared to GEX for CNBs and surgical specimens, respectively.

Biomarker (CNBs)	IHC/GEX	N	% Agreement	Kappa
**ER** (n = 168)	Pos/Pos	134	85.71%	0.395
	Neg/Neg	10		
	Pos/Neg	23		
	Neg/Pos	1		
**PgR** (n = 167)	Pos/Pos	110	80.83%	0.484
	Neg/Neg	25		
	Pos/Neg	19		
	Neg/Pos	13		
**HER2** (n = 166)	Pos/Pos	8	96.64%	0.708
	Neg/Neg	152		
	Pos/Neg	3		
	Neg/Pos	3		
**Ki67** (n = 166)	High/High	43	47.59%	0.152
	Low/Low	36		
	High/Low	84		
	Low/High	3		
**Subtype** (n = 150)	LumA/LumA	23	57.33%	0.264
	LumB/LumB	52		
	HER2/HER2	6		
	TNBC/Basal	5		
	Discordant subtype	64		
**Biomarker (Surgical)**	**IHC/GEX**	**N**	**% Agreement**	**Kappa**
**ER** (n = 213)	Pos/Pos	176	91.54%	0.636
	Neg/Neg	19		
	Pos/Neg	18		
	Neg/Pos	0		
**PgR** (n = 212)	Pos/Pos	153	86.32%	0.588
	Neg/Neg	30		
	Pos/Neg	16		
	Neg/Pos	13		
**HER2** (n = 216)	Pos/Pos	14	93.52%	0.632
	Neg/Neg	188		
	Pos/Neg	3		
	Neg/Pos	11		
**Ki67** (n = 215)	High/High	83	55.34%	0.205
	Low/Low	36		
	High/Low	99		
	Low/High	0		
**Subtype** (n = 203)	LumA/LumA	24	61.58%	0.398
	LumB/LumB	79		
	HER2/HER2	11		
	TNBC/Basal	11		
	Discordant subtype	78		

## Data Availability

RNA-sequencing-based gene expression data for SCAN-B cohort is available at Mendeley Data as a publicly accessible dataset [54]. Raw sequencing data is regarded personal information and by Swedish law cannot be made publicly accessible.

## Supplementary Materials

The following supporting information can be downloaded at: https://www.mdpi.com/article/10.3390/cancers14164000/s1, Table S1: Distribution of biomarker expression analyzed with IHC and GEX in CNBs and surgical specimens respectively; Table S2: Significantly Differentially Expressed Genes Between Paired CNBs and Surgical Specimen; Table S3: GSEA; Hallmarks of Cancer Database FDR q-val 0.1; Table S4: GSEA; Reactome Database FDR q-val 0.1.

## References

[1] Kooistra B., Wauters C., Strobbe L., Wobbes T. (2010). Preoperative cytological and histological diagnosis of breast lesions: A critical review. Eur. J. Surg. Oncol..

[2] Parker S.H., Burbank F., Jackman R.J., Aucreman C.J., Cardenosa G., Cink T.M., Coscia J.L., Eklund G.W., Evans W.P., Garver P.R. (1994). Percutaneous large-core breast biopsy: A multi-institutional study. Radiology.

[3] Dahlstrom J.E., Jain S., Sutton T., Sutton S. (1996). Diagnostic accuracy of stereotactic core biopsy in a mammographic breast cancer screening programme. Histopathology.

[4] Verkooijen H.M., Peeters P.H., Buskens E., Koot V.C., Borel Rinkes I.H., Mali W.P., van Vroonhoven T.J. (2000). Diagnostic accuracy of large-core needle biopsy for nonpalpable breast disease: A meta-analysis. Br. J. Cancer.

[5] Hoorntje L.E., Peeters P.H., Borel Rinkes I.H., Verkooijen H.M., Pijnappel R.M., Mali W.P. (2002). Stereotactic large core needle biopsy for all nonpalpable breast lesions?. Breast Cancer Res. Treat..

[6] Verkooijen H.M. (2002). Diagnostic accuracy of stereotactic large-core needle biopsy for nonpalpable breast disease: Results of a multicenter prospective study with 95% surgical confirmation. Int. J. Cancer.

[7] Dillon M.F., Hill A.D., Quinn C.M., O’Doherty A., McDermott E.W., O’Higgins N. (2005). The accuracy of ultrasound, stereotactic, and clinical core biopsies in the diagnosis of breast cancer, with an analysis of false-negative cases. Ann. Surg..

[8] (2020). Cancercentrum: Nationellt Kvalitetsregister för Bröstcancer (NKBC). https://statistik.incanet.se/brostcancer/.

[9] Burstein H.J., Curigliano G., Loibl S., Dubsky P., Gnant M., Poortmans P., Colleoni M., Denkert C., Piccart-Gebhart M., Regan M. (2019). Estimating the benefits of therapy for early-stage breast cancer: The St. Gallen International Consensus Guidelines for the primary therapy of early breast cancer 2019. Ann. Oncol..

[10] Masuda N., Lee S.J., Ohtani S., Im Y.H., Lee E.S., Yokota I., Kuroi K., Im S.A., Park B.W., Kim S.B. (2017). Adjuvant Capecitabine for Breast Cancer after Preoperative Chemotherapy. N. Engl. J. Med..

[11] von Minckwitz G., Huang C.S., Mano M.S., Loibl S., Mamounas E.P., Untch M., Wolmark N., Rastogi P., Schneeweiss A., Redondo A. (2019). Trastuzumab Emtansine for Residual Invasive HER2-Positive Breast Cancer. N. Engl. J. Med..

[12] Tutt A.N.J., Garber J.E., Kaufman B., Viale G., Fumagalli D., Rastogi P., Gelber R.D., de Azambuja E., Fielding A., Balmaña J. (2021). Adjuvant Olaparib for Patients with BRCA1- or BRCA2-Mutated Breast Cancer. N. Engl. J. Med..

[13] EBCTCG (2018). Long-term outcomes for neoadjuvant versus adjuvant chemotherapy in early breast cancer: Meta-analysis of individual patient data from ten randomised trials. Lancet Oncol..

[14] Robertson S., Rönnlund C., de Boniface J., Hartman J. (2019). Re-testing of predictive biomarkers on surgical breast cancer specimens is clinically relevant. Breast Cancer Res. Treat..

[15] Greer L.T., Rosman M., Mylander W.C., Hooke J., Kovatich A., Sawyer K., Buras R.R., Shriver C.D., Tafra L. (2013). Does breast tumor heterogeneity necessitate further immunohistochemical staining on surgical specimens?. J. Am. Coll. Surg..

[16] Zell J.A., Tsang W.Y., Taylor T.H., Mehta R.S., Anton-Culver H. (2009). Prognostic impact of human epidermal growth factor-like receptor 2 and hormone receptor status in inflammatory breast cancer (IBC): Analysis of 2,014 IBC patient cases from the California Cancer Registry. Breast Cancer Res..

[17] Denkert C., Loibl S., Müller B.M., Eidtmann H., Schmitt W.D., Eiermann W., Gerber B., Tesch H., Hilfrich J., Huober J. (2013). Ki67 levels as predictive and prognostic parameters in pretherapeutic breast cancer core biopsies: A translational investigation in the neoadjuvant GeparTrio trial. Ann. Oncol..

[18] Harris L.N., Ismaila N., McShane L.M., Andre F., Collyar D.E., Gonzalez-Angulo A.M., Hammond E.H., Kuderer N.M., Liu M.C., Mennel R.G. (2016). Use of Biomarkers to Guide Decisions on Adjuvant Systemic Therapy for Women with Early-Stage Invasive Breast Cancer: American Society of Clinical Oncology Clinical Practice Guideline. J. Clin. Oncol..

[19] Roepman P., Horlings H.M., Krijgsman O., Kok M., Bueno-de-Mesquita J.M., Bender R., Linn S.C., Glas A.M., van de Vijver M.J. (2009). Microarray-based determination of estrogen receptor, progesterone receptor, and HER2 receptor status in breast cancer. Clin. Cancer Res..

[20] Wilson T.R., Xiao Y., Spoerke J.M., Fridlyand J., Koeppen H., Fuentes E., Huw L.Y., Abbas I., Gower A., Schleifman E.B. (2014). Development of a robust RNA-based classifier to accurately determine ER, PR, and HER2 status in breast cancer clinical samples. Breast Cancer Res. Treat..

[21] Rantalainen M., Klevebring D., Lindberg J., Ivansson E., Rosin G., Kis L., Celebioglu F., Fredriksson I., Czene K., Frisell J. (2016). Sequencing-based breast cancer diagnostics as an alternative to routine biomarkers. Sci. Rep..

[22] Bastani M., Vos L., Asgarian N., Deschenes J., Graham K., Mackey J., Greiner R. (2013). A machine learned classifier that uses gene expression data to accurately predict estrogen receptor status. PLoS ONE.

[23] Brueffer C., Vallon-Christersson J., Grabau D., Ehinger A., Häkkinen J., Hegardt C., Malina J., Chen Y., Bendahl P.O., Manjer J. (2018). Clinical Value of RNA Sequencing-Based Classifiers for Prediction of the Five Conventional Breast Cancer Biomarkers: A Report from the Population-Based Multicenter Sweden Cancerome Analysis Network-Breast Initiative. JCO Precis. Oncol..

[24] Cardoso F., van’t Veer L.J., Bogaerts J., Slaets L., Viale G., Delaloge S., Pierga J.Y., Brain E., Causeret S., DeLorenzi M. (2016). 70-Gene Signature as an Aid to Treatment Decisions in Early-Stage Breast Cancer. N. Engl. J. Med..

[25] Gnant M., Filipits M., Greil R., Stoeger H., Rudas M., Bago-Horvath Z., Mlineritsch B., Kwasny W., Knauer M., Singer C. (2014). Predicting distant recurrence in receptor-positive breast cancer patients with limited clinicopathological risk: Using the PAM50 Risk of Recurrence score in 1478 postmenopausal patients of the ABCSG-8 trial treated with adjuvant endocrine therapy alone. Ann. Oncol..

[26] Filipits M., Nielsen T.O., Rudas M., Greil R., Stöger H., Jakesz R., Bago-Horvath Z., Dietze O., Regitnig P., Gruber-Rossipal C. (2014). The PAM50 risk-of-recurrence score predicts risk for late distant recurrence after endocrine therapy in postmenopausal women with endocrine-responsive early breast cancer. Clin. Cancer Res..

[27] Sparano J.A., Gray R.J., Ravdin P.M., Makower D.F., Pritchard K.I., Albain K.S., Hayes D.F., Geyer C.E., Dees E.C., Goetz M.P. (2019). Clinical and Genomic Risk to Guide the Use of Adjuvant Therapy for Breast Cancer. N. Engl. J. Med..

[28] Paik S., Shak S., Tang G., Kim C., Baker J., Cronin M., Baehner F.L., Walker M.G., Watson D., Park T. (2004). A multigene assay to predict recurrence of tamoxifen-treated, node-negative breast cancer. N. Engl. J. Med..

[29] Parker J.S., Mullins M., Cheang M.C., Leung S., Voduc D., Vickery T., Davies S., Fauron C., He X., Hu Z. (2009). Supervised risk predictor of breast cancer based on intrinsic subtypes. J. Clin. Oncol..

[30] Saal L.H., Vallon-Christersson J., Häkkinen J., Hegardt C., Grabau D., Winter C., Brueffer C., Tang M.H., Reuterswärd C., Schulz R. (2015). The Sweden Cancerome Analysis Network—Breast (SCAN-B) Initiative: A large-scale multicenter infrastructure towards implementation of breast cancer genomic analyses in the clinical routine. Genome Med..

[31] Rydén L., Loman N., Larsson C., Hegardt C., Vallon-Christersson J., Malmberg M., Lindman H., Ehinger A., Saal L.H., Borg Å. (2018). Minimizing inequality in access to precision medicine in breast cancer by real-time population-based molecular analysis in the SCAN-B initiative. Br. J. Surg..

[32] (2020). Cancercentrum R: Kvalitetsbilaga för Bröstpatologi (KVAST-bilaga). https://kunskapsbanken.cancercentrum.se/diagnoser/brostcancer/vardprogram/kvalitetsdokument-for--patologi/.

[33] Goldhirsch A., Winer E.P., Coates A.S., Gelber R.D., Piccart-Gebhart M., Thürlimann B., Senn H.J. (2013). Personalizing the treatment of women with early breast cancer: Highlights of the St Gallen International Expert Consensus on the Primary Therapy of Early Breast Cancer 2013. Ann. Oncol..

[34] Nielsen T.O., Leung S.C.Y., Rimm D.L., Dodson A., Acs B., Badve S., Denkert C., Ellis M.J., Fineberg S., Flowers M. (2021). Assessment of Ki67 in Breast Cancer: Updated Recommendations from the International Ki67 in Breast Cancer Working Group. J. Natl. Cancer Inst..

[35] Tusher V.G., Tibshirani R., Chu G. (2001). Significance analysis of microarrays applied to the ionizing radiation response. Proc. Natl. Acad. Sci. USA.

[36] Subramanian A., Tamayo P., Mootha V.K., Mukherjee S., Ebert B.L., Gillette M.A., Paulovich A., Pomeroy S.L., Golub T.R., Lander E.S. (2005). Gene set enrichment analysis: A knowledge-based approach for interpreting genome-wide expression profiles. Proc. Natl. Acad. Sci. USA.

[37] Usami S., Moriya T., Kasajima A., Suzuki A., Ishida T., Sasano H., Ohuchi N. (2005). Pathological aspects of core needle biopsy for non-palpable breast lesions. Breast Cancer.

[38] Cahill R.A., Walsh D., Landers R.J., Watson R.G. (2006). Preoperative profiling of symptomatic breast cancer by diagnostic core biopsy. Ann. Surg. Oncol..

[39] Usami S., Moriya T., Amari M., Suzuki A., Ishida T., Sasano H., Ohuchi N. (2007). Reliability of prognostic factors in breast carcinoma determined by core needle biopsy. Jpn. J. Clin. Oncol..

[40] Burge C.N., Chang H.R., Apple S.K. (2006). Do the histologic features and results of breast cancer biomarker studies differ between core biopsy and surgical excision specimens?. Breast.

[41] Connor C.S., Tawfik O.W., Joyce A.J., Davis M.K., Mayo M.S., Jewell W.R. (2002). A comparison of prognostic tumor markers obtained on image-guided breast biopsies and final surgical specimens. Am. J. Surg..

[42] Di Loreto C., Puglisi F., Rimondi G., Zuiani C., Anania G., Della Mea V., Beltrami C.A. (1996). Large core biopsy for diagnostic and prognostic evaluation of invasive breast carcinomas. Eur. J. Cancer.

[43] Mann G.B., Fahey V.D., Feleppa F., Buchanan M.R. (2005). Reliance on hormone receptor assays of surgical specimens may compromise outcome in patients with breast cancer. J. Clin. Oncol..

[44] Romero Q., Bendahl P.O., Klintman M., Loman N., Ingvar C., Rydén L., Rose C., Grabau D., Borgquist S. (2011). Ki67 proliferation in core biopsies versus surgical samples—A model for neo-adjuvant breast cancer studies. BMC Cancer.

[45] Hadad S.M., Jordan L.B., Roy P.G., Purdie C.A., Iwamoto T., Pusztai L., Moulder-Thompson S.L., Thompson A.M. (2016). A prospective comparison of ER, PR, Ki67 and gene expression in paired sequential core biopsies of primary, untreated breast cancer. BMC Cancer.

[46] Orozco J.I.J., Chang S.C., Matsuba C., Ensenyat-Mendez M., Grunkemeier G.L., Marzese D.M., Grumley J.G. (2021). Is the 21-Gene Recurrence Score on Core Needle Biopsy Equivalent to Surgical Specimen in Early-Stage Breast Cancer? A Comparison of Gene Expression Between Paired Core Needle Biopsy and Surgical Specimens. Ann. Surg. Oncol..

[47] Qi P., Yang Y., Bai Q.M., Xue T., Ren M., Yao Q.L., Yang W.T., Zhou X.Y. (2021). Concordance of the 21-gene assay between core needle biopsy and resection specimens in early breast cancer patients. Breast Cancer Res. Treat..

[48] Valachis A., Mauri D., Polyzos N.P., Chlouverakis G., Mavroudis D., Georgoulias V. (2011). Trastuzumab combined to neoadjuvant chemotherapy in patients with HER2-positive breast cancer: A systematic review and meta-analysis. Breast.

[49] Gianni L., Eiermann W., Semiglazov V., Manikhas A., Lluch A., Tjulandin S., Zambetti M., Vazquez F., Byakhow M., Lichinitser M. (2010). Neoadjuvant chemotherapy with trastuzumab followed by adjuvant trastuzumab versus neoadjuvant chemotherapy alone, in patients with HER2-positive locally advanced breast cancer (the NOAH trial): A randomised controlled superiority trial with a parallel HER2-negative cohort. Lancet.

[50] Korde L.A., Somerfield M.R., Carey L.A., Crews J.R., Denduluri N., Hwang E.S., Khan S.A., Loibl S., Morris E.A., Perez A. (2021). Neoadjuvant Chemotherapy, Endocrine Therapy, and Targeted Therapy for Breast Cancer: ASCO Guideline. J. Clin. Oncol..

[51] (2020). Cancercentrum R: Nationellt Vårdprogram Bröstcancer. https://cancercentrum.se/samverkan/cancerdiagnoser/brost/vardprogram/.

[52] Acs B., Leung S.C.Y., Kidwell K.M., Arun I., Augulis R., Badve S.S., Bai Y., Bane A.L., Bartlett J.M.S., Bayani J. (2022). Systematically higher Ki67 scores on core biopsy samples compared to corresponding resection specimen in breast cancer: A multi-operator and multi-institutional study. Mod. Pathol..

[53] Smith I., Robertson J., Kilburn L., Wilcox M., Evans A., Holcombe C., Horgan K., Kirwan C., Mallon E., Sibbering M. (2020). Long-term outcome and prognostic value of Ki67 after perioperative endocrine therapy in postmenopausal women with hormone-sensitive early breast cancer (POETIC): An open-label, multicentre, parallel-group, randomised, phase 3 trial. Lancet Oncol..

[54] Vallon-Christersson J. (2022). RNA Sequencing-Based Single Sample Predictors of Molecular Subtype and Risk of Recurrence for Clinical Assessment of Early-Stage Breast Cancer. Mendeley Data V1. https://data.mendeley.com/datasets/yzxtxn4nmd.

